# Synthetic Fluorophores for Visualizing Biomolecules in Living Systems

**Published:** 2016

**Authors:** V. I. Martynov, A. A. Pakhomov, N. V. Popova, I. E. Deyev, A. G. Petrenko

**Affiliations:** Shemyakin–Ovchinnikov Institute of Bioorganic Chemistry, Russian Academy of Sciences, Miklukho-Maklaya St., 16/10, Moscow, 117997, Russia

**Keywords:** fluorophore, fluorescence microscopy, site-directed reaction, measurement of ion concentration, measurement of local pH

## Abstract

The last decade has witnessed significant advance in the imaging of living systems using fluorescent markers. This progress has been primarily associated with the discovery of different spectral variants of fluorescent proteins. However, the fluorescent protein technology has its own limitations and, in some cases, the use of low-molecular-weight fluorophores is preferable. In this review, we describe the arsenal of synthetic fluorescent tools that are currently in researchers’ hands and span virtually the entire spectrum, from the UV to visible and, further, to the near-infrared region. An overview of recent advances in site-directed introduction of synthetic fluorophores into target cellular objects is provided. Application of these fluorescent probes to the solution of a wide range of biological problems, in particular, to the determination of local ion concentrations and pH in living systems, is discussed.

## INTRODUCTION

Fluorescence-based molecular markers have for a long time served as a tool for *in vitro *imaging of biomolecules. Fluorescent labeling with a synthetic fluorophore was first reported in 1942, when fluorescein isothiocyanate (FITC)-labeled anti-pneumococcal antibodies were obtained [1]. Until the 1980s, fluorescent labeling was mostly used to analyze fixed biological specimens. Over the past two decades, a number of methods have been designed that allow one to insert fluorescent tags into living objects [2], in particularly, as genetically encoded chimeras of target cellular proteins (TCPs) with GFP-like fluorescent proteins (FPs) [3-5]. However, in some cases, the analysis of living systems requires the use of low-molecular-weight fluorescent probes [6, 7] to directly modify TCP [8, 9]. The main advantage of these fluorophores is their small size and the availability of compounds with the desired chemical and photophysical properties.

The possibility of using a certain fluorophore depends on its chemical (reactivity, solubility, lipophilic properties, p*K*a, and stability) and photophysical properties (excitation maximum (λ_ex_), emission maximum (λ_em_), extinction coefficient (ε), quantum yield (Φ), lifetime of the excited state, and photostability). The extinction coefficient multiplied by the quantum yield (ε × Φ) is the universal parameter used to determine the sensitivity of this method for different fluorophores. This value is directly proportional to the brightness and takes into account the amount of absorbed light and the yield of fluorophore emission.

## PROPERTIES OF SYNTHETIC FLUOROPHORES

**Fluorophores emitting in the UV and blue spectral ranges**

**Fig. 1:**
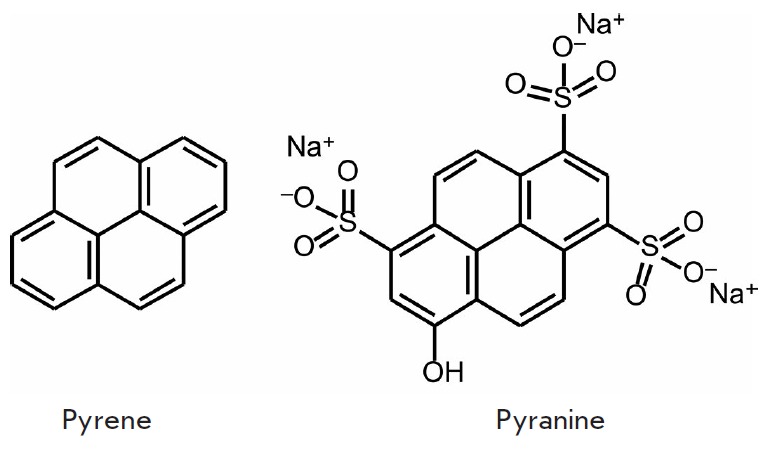
Fluorophores based on condensed aromatic compounds with emission in the UV- and blue spectral ranges

Fluorophores emitting in the UV spectral range are used to label living systems not that frequently, since UV light is toxic for them. Furthermore, it is difficult to distinguish between the fluorescence signals of these tags and cell autofluorescence. Pyrene derivatives (*Fig. 1*) are a classic example of fluorophores emitting in the near-UV spectral range: they are characterized by λ_ex_ = 340 nm, λ_em_ = 376 nm, a high quantum yield Φ = 0.75, chemical stability, and long fluorescence lifetime, which allows fluorophore molecules to form excimers with a bathochromic shift in the emission spectra. These properties of pyrenes are used in the monitoring of conformational changes in the protein structure [10] and to determine the concentrations of ions of certain metals [11, 12]. Pyrene derivatives, such as 8-hydroxy-1,3,6-pyrenetrisulfonate (pyranine, *Fig. 1*), are used as pH indicators or sensors for Cu^+^ ions [13]. The 8-O-carboxymethylpyranine derivative is characterized by λex/λem 401.5/428.5 nm and ε = 2.5 × 10^4^ M^-1^cm^-1^ (405 nm). This fluorophore can be used as a bright and photostable tag emitting in the violet spectral range for multicolor labeling of cellular objects [14].

**Fig. 2:**
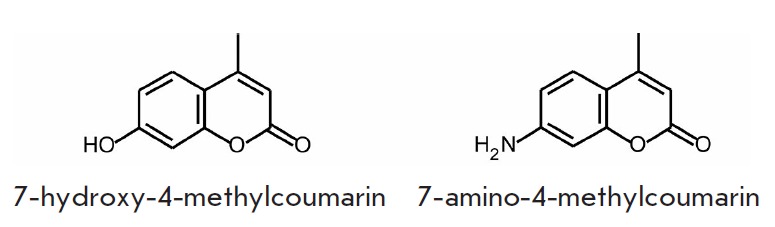
Fluorophores based on coumarin derivatives

Fluorescent markers based on coumarin derivatives are widely employed as chemosensors and in the labeling of biomolecules [15, 16]. A substituent inserted at position 7 of coumarin yields fluorophores emitting in the visible range of the spectrum: e.g., 7-hydroxy- 4-methylcoumarin (*Fig. 2*). This fluorophore is characterized by λ_ex_ = 360 nm, λ_em_ = 450 nm, ε = 1.7 × 10^3^ M^-1^cm^-1^, and Φ = 0.63. 7-Hydroxycoumarin derivatives act as an intracellular fluorescent sensor of phosphatase activity; its mixed carbonates are used to determine the lipase and esterase activities [17, 18]. A related compound, 7-amino-4-methylcoumarin (*Fig. 2*), exhibits the same spectral properties as the hydroxy derivative at pH > 5.

**Fig. 3:**
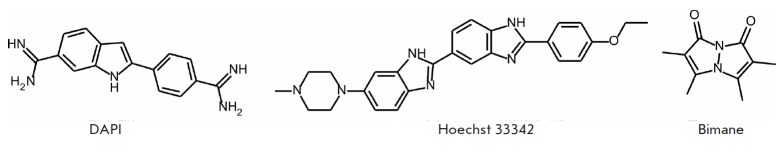
Synthetic fluorophores emitting in the blue and cyan spectral ranges

The indole derivative, 4’,6-diamidino-2-phenylindole (DAPI; *Fig. 3*), was first synthesized in 1971 at the Otto Dann’s laboratory in pursuit of anti-trypanosomiasis drugs. This compound proved inefficient as medication but demonstrated DNA-binding ability [19]. Since the binding of DAPI to DNA significantly increases fluorescence in the blue spectral range (λ_em_ = 461 nm for DAPI bound to DNA), this marker is widely used to label DNA in living cells [20]. It has recently been shown that irradiation of DAPI with UV light or laser light with λ = 405 nm results in its photoconversion [21, 22]. The emission maximum of the fluorophore shifts toward the green region of the spectrum (505 nm) after argon laser excitation of the photoconverted form of DAPI at 458 nm. Furthermore, the photoconverted green form of the fluorophore loses its color after blue-light irradiation [22]. This property was used in single-molecule localization microscopy (the SMLM method in subdiffraction imaging) of DNA, which has made it possible to reconstruct the accurate map of distribution of these molecules in cell nuclei and chromosomes during mitosis [20].

The fluorescent dibenzimidazole derivatives were first synthesized and used by Hoechst AG company for fluorescence microscopy. The compound Hoechst 33342 (*Fig. 3*) fluoresces in the cyan-blue spectral range and has an emission maximum at 461 nm. It binds to DNA, easily penetrates through the cell membrane, and can be used for experiments on living cells [20].

Bimane, 1,5-diazabicyclo[3.3.0]octa-3,6-dien-2,8- dione (*Fig. 3*), is characterized by λ_ex_ = 390 nm, λ_em_ = 482 nm, and Φ = 0.3. Bimane fluorescence is degree of quenching depends on the distance between these two residues (≤ 10–15 nm). This property of the fluorophore was used for real-time detection of the conformational changes of enzymes during substrate binding [23, 24].

**Fluorophores emitting in the green–yellow spectral range**

**Fig. 4:**
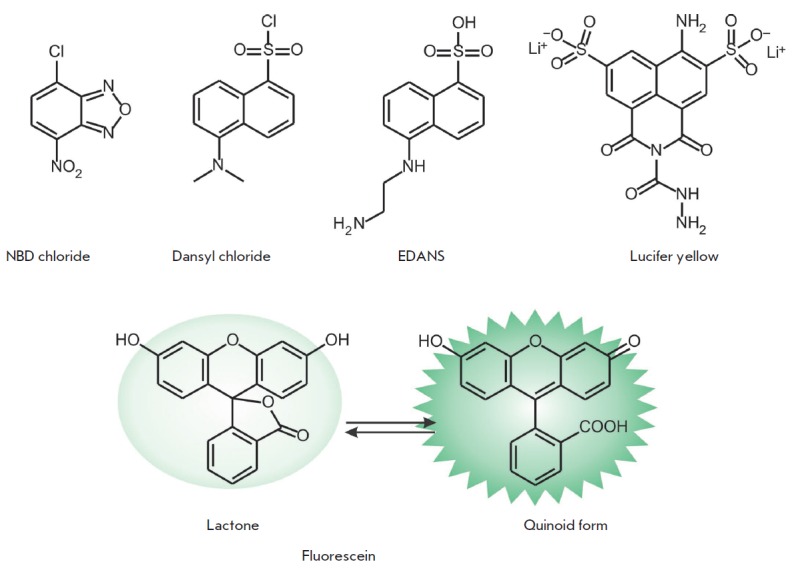
Fluorophores emitting in the green-yellow spectral range

NBD (4-nitrobenzo-2-oxa-1,3-diazole) and its derivatives exhibit emission in the green region of the spectrum. NBD chloride (*Fig. 4*) reacts with amino and thiol groups. Complexes between NBD chloride and primary amines have the excitation and emission maxima λ_ex_ = 465 nm, λ_em_ = 535 nm (ε = 2.2 × 10^4^ M^-1^cm^-1^ and Φ = 0.3). Another NBD derivative that selectively interacts with cysteine has been successfully used as a fluorescent sensor of Cys in HeLa cells [25]. The sensitivity of NBD derivatives to the microenvironment proved important in producing lipid markers [26, 27] or new kinase substrates [28]. NBD-based Cu^2+^ and S^2-^ sensors that allow one to determine the concentration of these ions in a living cell have been designed [29].

NBD-SCN was used to detect cysteine and homocysteine. Substitution of the thiocyanate group with cysteine or homocysteine increases the intensity of NBD fluorescence at 550 nm 470- and 745-fold, respectively [30]. Moreover, NBD-SCN exhibits relatively high membrane-penetrating properties and can be used to visualize changes in the concentration of cysteine and homocysteine in a living cell [30].

Naphthalene derivatives are among the most frequently used fluorophores emitting in the green spectral range. This group of tags includes dansyl chloride that reacts with amino groups and EDANS (*Fig. 4*). Derivatives of this compound are characterized by λ_ex_ = 336 nm, λ_em_ = 520 nm, ε = 6.1 × 10^3^ M^-1^cm^-1^, and Φ = 0.27. EDANS-based fluorescent markers are currently used in *in vivo *experiments [31]. Another fluorophore, 4-amino- 3,6-disulfonylnaphthalimide, is characterized by fluorescence emission in the yellow spectral range. Its carbohydrazide, known as Lucifer yellow (λ_ex_ = 428 nm, λ_em_ = 534 nm, *Fig. 4*), is used as a polar label and in twophoton excitation experiments [32].

Fluorophore fluorescein (*Fig. 4*) exhibits unique properties: it can exist in aqueous solutions as seven prototropic forms, including the most biologically important ones – mono-anionic and dianionic forms – which interconvert with p*K*_a_ ~ 6.4 [33]. The dianionic form displays maximal fluorescence (λ_ex_ = 490 nm, λ_em_ = 514 nm, ε = 9.3 × 10^4^ M^-1^cm^-1^, and Φ = 0.95). The pH sensitivity of fluorescein derivatives was used to produce fluorescent pH indicators [34, 35]. These derivatives were employed to obtain sensors for ions of various metals: e.g., Fluo-3 for measuring the concentration of calcium ions in living cells [36, 37]. Fluorescein exists in two equilibrium forms: as lactone and the quinoid form (*Fig. 4*). Acylation or alkylation of phenol groups results in the fixation of a molecule as nonfluorescent lactone, which can be used to synthesize fluorogenic substrates for a number of enzymes [38, 39]. However, fluorescein-based fluorophores have significant drawbacks, as well. They are characterized by a high rate of photobleaching; the wide emission band of these fluorophores limits their application in multi-color labeling of cellular objects. Furthermore, they are prone to self-quenching at high densities of tag insertion in TCP.

**Fig. 5:**
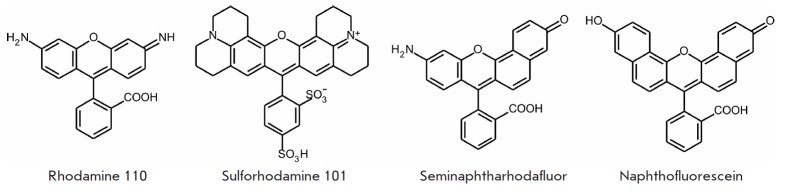
Xanthene-based synthetic fluorophores

Another group of synthetic fluorophores emitting in the green spectral range is based on rhodamine derivatives. Introduction of various substituents into the rhodamine structure allows one to tune its spectral characteristics. The most typical example is rhodamine 110 (*Fig. 5*) (λ_ex_ = 497 nm, λ_em_ = 520 nm, ε = 7.6 × 10^4^ M^-1^cm^-1^, and Φ = 0.88 [40]). Insertion of four-membered azetidine rings at two nitrogen atoms substantially increases the quantum yield and brightness of the fluorophore [41], while insertion of four methyl groups at the N, N’ atoms shifts the excitation and emission maxima towards the long-wavelength region (λ_ex_/ λ_em_ 548/572 nm), but reduces the quantum yield of the fluorophore (Φ = 0.41) in aqueous solutions [42]. Rhodamines containing rigid cyclic systems instead of amino groups are characterized by higher quantum yields; their spectra are shifted towards the long-wavelength region. In particular, sulforhodamine 101 (Texas Red) (*Fig. 5*) and its derivatives – the fluorophores that are most frequently used in cellular biology [43, 44] – are also used as photosensitizers in photodynamic therapy [45]. Together with fluorescein, rhodamine tags are components of FRET pairs [46, 47]. Substitution of both amino groups in rhodamine can yield its nonfluorescent derivative. This property is used in the synthesis of photoactivable rhodamine analogues [48] and to synthesize fluorogenic substrates when studying the mechanisms of enzyme catalysis. Rhodamine 110 derivatives have been used as substrates to determine the activity of various enzymes [49]. Hybrid fluorophores consisting of a polypeptide-linked quantum dot and rhodamine that can be cleaved by caspase-1 have been used in apoptosis assays [50]. Rhodamine derivatives are also used when designing indicators of pH and the ions of some metals [51, 52].

The compounds under the Alexa Fluor trademark are a large group of hydrophilic negatively charged tags that are represented by sulfated derivatives of various fluorophores, such as fluorescein, coumarin, cyanine, or rhodamine. The well-known rhodamine derivative Alexa Fluor 488 exhibits properties largely similar to those of FITC (λ_ex_ = 493 nm, λ_em_ = 519 nm). However, unlike FITC, Alexa Fluor 488 is characterized by higher photostability, higher brightness, and lower pH sensitivity. Optimal results in comparable experiments on specific labeling of modified histones were demonstrated by Fab fragments labeled with Alexa Fluor 488 [53]. Alexa Fluor 488 can act as a donorfluorophore to study the structure of various cellular receptors using the FRET effect [54].

**Fluorophores emitting in the red, farred, and near-infrared spectral range**

Fluorophores emitting in the far-red and near-infrared spectral ranges are of greatest interest, since the lightexciting fluorescence of these fluorophores is non-toxic to living systems. Furthermore, infrared rays can penetrate living tissues much deeper than shorter wave light. In addition, background autofluorescence has virtually no effect on the imaging of biomolecules in living systems using far-red and infrared fluorophores. Unfortunately, the majority of known synthetic fluorophores belonging to this group have a significant drawback: a low quantum yield of fluorescence in aqueous solutions. Probably, among the fluorophores of this group, special attention should be focused on fluorescein and rhodamine derivatives with xanthene structures modified by the addition of aromatic rings. These substituents cause a significant bathochromic shift in fluorescence spectra. One of these derivatives, naphthofluorescein (*Fig. 5*), fluoresces in alkaline solutions at much longer wavelengths (λ_ex_/λ_em_ – 595/660 nm). However, all the advantages of this far-red fluorescent tag are outweighed by the fact that it has a lower extinction coefficient (ε = 4.4 × 10^4^ M^-1^cm^-1^) and quantum yield (Φ = 0.14). This series of derivatives has been successfully used as sensors in living cells [55, 56].

In efforts to obtain rhodamine derivatives absorbing at longer wavelengths, its analogues with the oxygen atom between two aromatic cycles substituted by silicon (Si-rhodamine), germanium (Ge-rhodamine), or tin (Snrhodamine) atoms were synthesized [57]. The resulting derivatives retain the key characteristics of rhodamine, such as a high quantum yield in aqueous solutions, resistance to photobleaching, and high water solubility. Three new compounds – SiR680, SiR700, and SiR720 – with fluorescence in the near-infrared region (670– 740 nm) were obtained by inserting additional aromatic substituents in Si-rhodamine. SiR680 and SiR700 were shown to exhibit appreciably high quantum yields in aqueous solutions (Φ = 0.35 and 0.12, respectively) [58]. Activated succinimide derivatives of SiR700 were used* in vivo *for the imaging of a tumor growth [58, 59].

Xanthene dyes with a structure containing an additional aromatic ring exhibit unique properties. Unlike the symmetric fluorescein and rhodamine derivatives, the resonance forms of these compounds are not equivalent to each other and have different spectral properties. Hence, the asymmetry of these tags can be used to design ratiometric fluorescent indicators. The ratio between the fluorescent intensities of different forms of indicators allows one to accurately determine the intracellular concentration of various ions. Seminaphthofluorescein- based fluorophores are used as pH sensors and indicators of other ions. Rhodol-based seminaphthoxanthenes are also applied as pH indicators. The ratiometric pH sensor seminaphthorhodafluor (*Fig. 5*) is one of such examples [60, 61]. This compound is characterized by λ_ex_ = 573 nm, λ_em_ = 631 nm, ε = 4.4 × 10^4^ M^-1^cm^-1^, and Φ = 0.092 at high pH values.

**Fig. 6:**
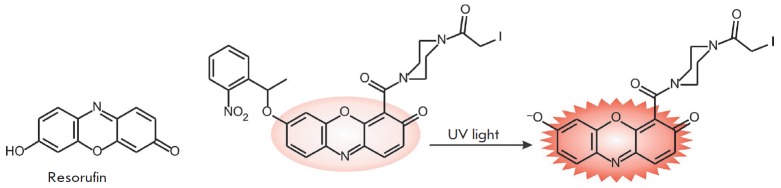
Resorufin and its photoactivatable *o*-nitrobenzyl derivative

Resorufin (*Fig. 6*) is used, in particular, for real-time detection of endogenous phosphatase activity in living cells [62]. At pH > 7.5, resorufin exists in anionic form with fluorescence emission in the red spectral range (λ_ex_ = 572 nm, λ_em_ = 585 nm, ε = 5.6 × 10^4^ M^-1^cm^-1^, and Φ = 0.74). The fluorescence intensity of this dye significantly decreases at low pH values.

Some synthetic fluorophores can be modified so that their fluorescence is “switched on” only after activation with light of a certain wavelength. These photoactivatable, or latent, fluorophores are used for space- and time-resolved dynamic imaging of processes requiring the activation of small populations of fluorescent markers. In particular, these fluorogenic markers are synthesized via reactions between a fluorophore and* o*-nitrobenzyl bromide. A molecule can be activated by irradiation at 365 nm; the *o*-nitrobenzyl group is cleaved to release the active fluorophore (*Fig. 6*). Active migration of microtubules during mitosis was demonstrated for the first time, and the dynamics of actin microfilaments was studied using photoactivation of a tubulin-conjugated fluorogenic probe [63]. A number of photoactivable coumarin analogues capable of penetrating into the cell have been synthesized [64, 65]. After penetrating into the cell, a small population of coumarin molecules was activated and used as a fluorescent reporter to monitor the migration of molecules through gap junctions [65].

Borodifluorodipyrromethene-based compounds widely known as BODIPY are used to synthesize fluorescent markers [66, 67], including those for labeling biomolecules in living cells [68]. They are characterized by high photostability and quantum yield, neutral charge, and narrow absorption and emission bands.

**Fig. 7:**
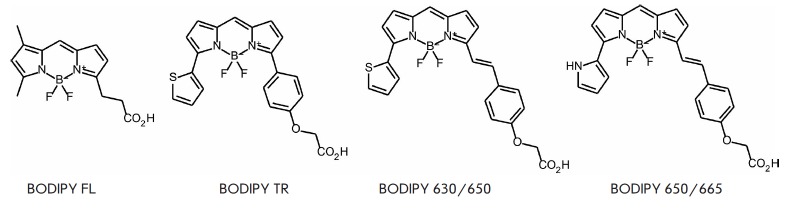
BODIPY-based fluorophores

This series of dyes can be tuned to the desired wavelength using certain substituents [69]. However, wide application of these fluorophores is limited because of their poor solubility in water. Some BODIPY fluorophores (*Fig. 7*) exhibit spectral properties similar to those of fluorescein: e.g., BODIPY FL (λ_ex_ = 505 nm, λ_em_ = 511 nm, ε = 9.1 × 10^4^ M^-1^cm^-1^, and Φ = 0.94). Insertion of additional aromatic substituents in the BODIPY FL molecule (*Fig. 7*) shifts emission towards the red and far-red regions (BODIPY TR, BODIPY 630/650 and BODIPY 650/665).

Most BODIPY-based labels are stable fluorescent markers. However, fluorophores altering their optical properties during photoactivation or upon binding to biologically important molecules have also been synthesized [70, 71]. A neutrally charged BODIPY can penetrate through the cell membrane. BODIPY and some of its derivatives exhibit appreciably high lipophilic properties and, therefore, are accumulated mostly in the membranes of subcellular structures [72]. Hence, modified BODIPY derivatives containing hydrophilic moieties are required for the imaging of biomolecules localized in cytosol.

BODIPY and its derivatives are characterized by a small Stokes shift, which is the reason for the selfquenching of these markers at a high density of biomolecule labeling. This property is used to synthesize fluorogenic substrates for proteinases whose fluorescence intensity increases during the proteolysis of proteins labeled with this tag with a higher density [73].

**Fig. 8:**
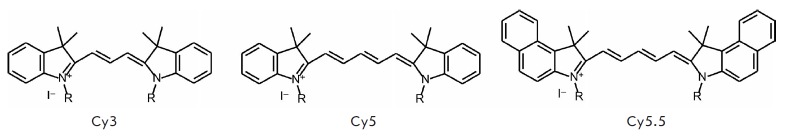
Derivatives of cyanine dyes Cy3, Cy5, and Cy5.5

Carbocyanine dyes (cyanins) are compounds with polymethine chains of different lengths that have an odd number of carbon atoms between two nitrogen atoms (R_2_N-(CH=CH)*_n_*-CH=N+R_2_) [70] (*Fig. 8*). The structure of these compounds is very similar to that of the chromophores in the visual pigment rhodopsin [74]. This property was recently used to design a construct encoding a specific protein binding retinoic acid (CRABPII) that can form a complex with the fluorogenic derivative of cyanine dye. Unlike the original profluorophore, this complex is characterized by a bright fluorescence in the far-red spectral range and high quantum yield [75]. Labels with only one terminal nitrogen atom involved in the aromatic heterocycle are known as hemicyanine dyes. Hemicyanines are used as ratiometric fluorescent pH sensors in *in vivo *experiments [76]. Cyanine tags in which the terminal chargecarrying atoms are directly bound to the methine chain are called streptocyanine tags. Streptocyanine dyes have been used as an indicator of superoxide dismutase activity [77].

Carbocyanine compounds are given names corresponding to the number of carbon atoms between the dihydroindole components of the molecule. In terms of its spectral characteristics, Cy3 (*Fig. 8*) is comparable to tetramethylrhodamine (λ_ex_ = 554 nm, λ_em_ = 568 nm). The spectra of Cy5 are shifted towards longer wavelengths (λ_ex_ = 652 nm, λ_em_ = 672 nm), while the more extensive constructs, such as Cy7, exhibit fluorescence emission in the near-infrared region (λ_ex_ = 755 nm, λ_em_ = 788 nm). Cyanines are characterized by a high extinction coefficient (up to 300, 000 M^-1^;1cm^-1^) and high solubility in water. Absorption and emission can be shifted towards longer wavelengths either by increasing the length of the polymethine chain or by inserting an aromatic moiety of terminal heterocyclic fragments. Increasing the length of the polymethine chain by two carbon atoms shifts the absorption maximum by ~100 nm, while insertion of the benzene ring to the terminal indole residue shifts absorption by ~30 nm [78]. Such structural modifications are denoted with a “.5” index: e.g., Cy5.5.

The *p*-nitrobenzoyl derivative of the heptacyanine fluorophore emitting in the near-infrared region was used as a ratiometric sensor of cysteine in mitochondria under oxidative stress. It has been demonstrated that this fluorophore can be used in living mice as a sensor of Cys [79] and glutathione levels in living cells [80].

## SITE-DIRECTED REACTIONS OF A SYNTHETIC FLUOROPHORE CONJUGATION TO TCP

**Covalent binding reactions**

**Fig. 9:**
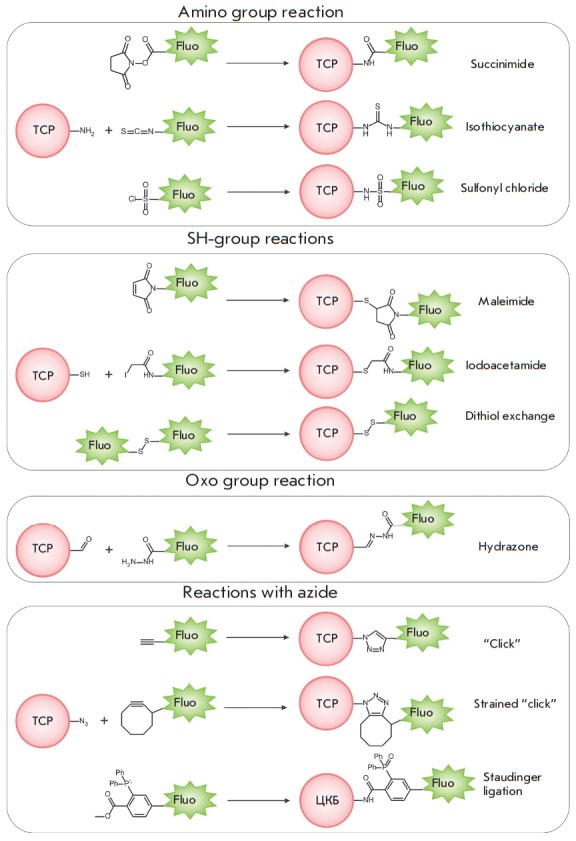
Reactions of covalent bond formation with biomolecules

Various chemical reactions are currently used to bind synthetic fluorophores to functional groups of biomolecules [81]. Succinimide ester (*Fig. 9*) is the most frequently used: its interaction with primary and secondary amino groups yields a stable amide bond. Isothiocyanate is another commonly used compound. Fluorophores modified with iodacetamide, maleimide, or dithioles are used to label sulfhydryl groups.

Bioorthogonal conjugation [82] and the so-called “click” chemistry [83-85] draw special attention. In this case, the chemical groups involved in the reaction of conjugation to a biomolecule do not react with other functional groups. These chemical groups are inserted in the molecules either via the metabolic machinery of the cell [86, 87] or due to the TCP enzymatic activity of [88, 89]. The azide moiety complies with all the requirements imposed on a bioorthogonal chemical group: it is characterized by high reactivity, selectivity, stability in aqueous media, and low reactivity towards biological molecule functional groups. Insertion of a small azide moiety only results in minor structural perturbations of the biomolecule. The bioorthogonal label inserted into a cellular object can be covalently bound to the fluorophore due to highly selective click reactions: Cu-catalyzed azide–alkyne cycloaddition is a classic example of those (*Fig. 9*) [90, 91]. However, Cu-catalyzed reactions can be mainly used in *in vitro *experiments, since the catalyst needs to be delivered to the reaction site in the living systems. In addition, copper is toxic at the concentrations used for labeling. Bertozzi et al. [92] have developed a method of modification where an alkyne is a component of the strained eight-membered ring (*Fig. 9*). In this system, the alkyne exhibits increased reactivity and does not require a catalyst. Later on, difluorocyclooctynes [93] with much higher reactivity were obtained, allowing one to use click chemistry for azide-labeled biomolecules in living organisms [94]. The Staudinger ligation is another example of bioorthogonal reaction application for *in vivo* labeling (*Fig. 9*) [95, 96].

**Reactions yielding sulfides and metalchelate fluorophore–TCP complexes**

**Fig. 10:**
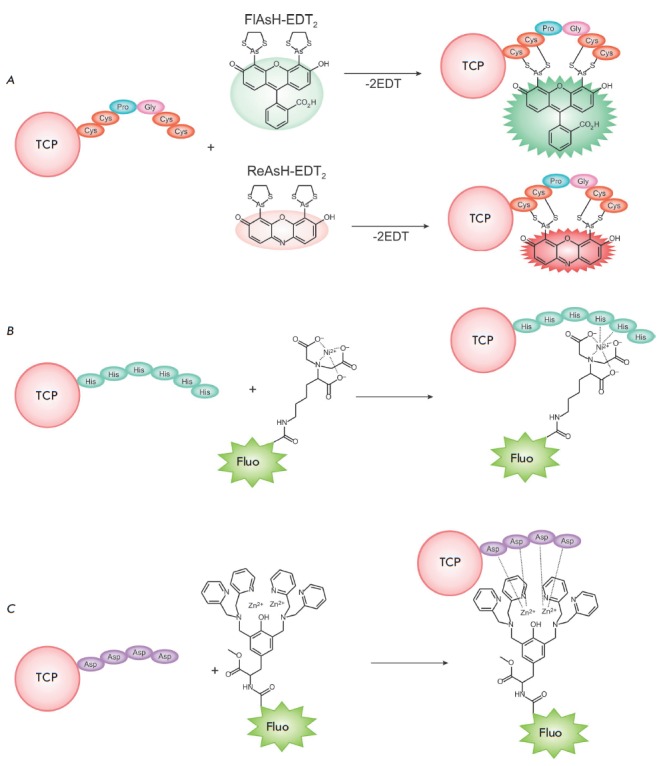
Reactions of sulfide and metal-chelate complex formation between a fluorophore and target cell protein (TCP)

Introduction of small amino acid sequences into the target protein is another promising approach in conjugating synthetic fluorophores to TCP. These sequences need to have an appreciably high affinity to the selected fluorescent marker. For example, the Cys- Cys-Pro-Gly-Cys-Cys sequence forms a hairpin-like structure due to the -Pro-Gly- insertion [97]. Hence, four cysteine residues form a cluster characterized by high affinity to organic arsenic compounds [98]. In particular, the arsenic-disubstituted derivative of fluorescein FlAsH (λ_ex_ = 508 nm, λ_em_ = 528 nm) reacts with this tetracysteine sequence to form a complex with a dissociation constant that lies in the picomolar range (*Fig. 10A*) [8, 99]. Furthermore, FlAsH exhibits bright green fluorescence only when bound to the tetracysteine sequence, thus significantly reducing background fluorescence. Besides FlAsH, there exists ReAsH (λ_ex_ = 593 nm, λ_em_ = 608 nm), a resorufin-based marker (*Fig. 10A*) exhibiting fluorescence in the red spectral range [8, 100].

It should be mentioned that FlAsH and ReAsH are membrane-permeable labels, which facilitates their delivery to the cell. Side reactions with monothiols are a drawback common to these compounds; however, nonspecific binding can be suppressed with an excess amount of dithiotreitol. Labeling with FlAsH and ReAsH is also complicated under oxidative conditions because of the oxidative reactions that the tetra-Cys sequence is involved in.

Metal coordination complexes are used in another method for fluorophore insertion into the TCP [101]. A polyhistidine sequence ((His)*n*, where *n *≥ 6) that forms complexes with nickel nitrile triacetate (Ni^2+^–NTA) acts as a complexing agent (*Fig. 10B*). Derivatives of cyanine dyes with one or two covalently bound Ni^2+^–NTA complexes were synthesized to specifically label proteins containing the poly-His-sequence. The disubstituted derivatives Cy3 and Cy5 have demonstrated a higher affinity compared to the monosubstituted ones and were used in FRET experiments to measure the distances in DNA complexes with the poly-His-labeled protein [102].

The key disadvantage of the poly-His/Ni^2+^–NTA system for *in vivo *experiments is the low binding affinity (the *K*d values lie within 1–20 μM), which negatively affects the stability of the fluorophore-TCP complex and, eventually, visualization of TCP. Piehler et al. synthesized fluorescein derivatives with 1–4 covalently bound NTA residues and characterized their interaction with the poly-His-sequence (His6 and His10). The stability of multivalent chelating groups to bind increased by more than four orders of magnitude, compared to that of mono-NTA and reached the subnanomolar level [103].

Poor permeability across the cell membrane is another serious limitation in using the Ni^2+^-NTA complex* in vivo*. Tampe et al. applied the membrane-translocating TAT-peptide (49RKKRRQRRR57) to deliver Ni^2+^-NTA inside the cell [104]. The resulting trisNTA/ His6-TAT49−57 complex was used to deliver fluorescently labeled NTA into the cell (the cytosol and nucleus); trisNTA is then predominantly bound to the His10-tagged intracellular protein. The translocating peptide His6-TAT49−57 was released, since it had a higher binding affinity to His10 (*K*_d_ = 0.1 nm) [103].

Sun et al. suggested a different approach to the synthesizing of membrane-permeable constructs [105]. They obtained a compound where NTA was covalently bound to fluorophore and aryl azide (Ni^2+^-NTA-AC). Ni^2+^-NTA-AC easily penetrated through the cell membrane and was bound to intracellular proteins carrying the poly-His tag. Light activation resulted in covalent binding of aryl azide to TCP, which increased fluorescence 13-fold and ensured stable binding to the fluorescent tag.

In addition to the tetracystein and poly-His sequences, poly-Asp ((Asp_4_)*_n_*, where *n *= 1–3) were also used to label TCP. Hamachi et al. synthesized fluorescein- tagged polynuclear Zn^2+^ complexes (a binuclear Zn^2+^ complex is shown in *Fig. 10C*) [106]. In this case, increased affinity was observed for a longer poly-Asp chain. Tetranuclear Zn^2+^ complexes were used for fluorescent labeling of the muscarinic acetylcholine receptor, when its initial activity was retained.

**Site-directed labeling using enzyme reactions**

Another technology of fluorophore insertion in TCP is based on enzyme reactions. The so-called SNAP-tag [107], CLIP-tag [108], HALO-tag [109], and TMP-tag [110-112] methods are used in this case.

**Fig. 11:**
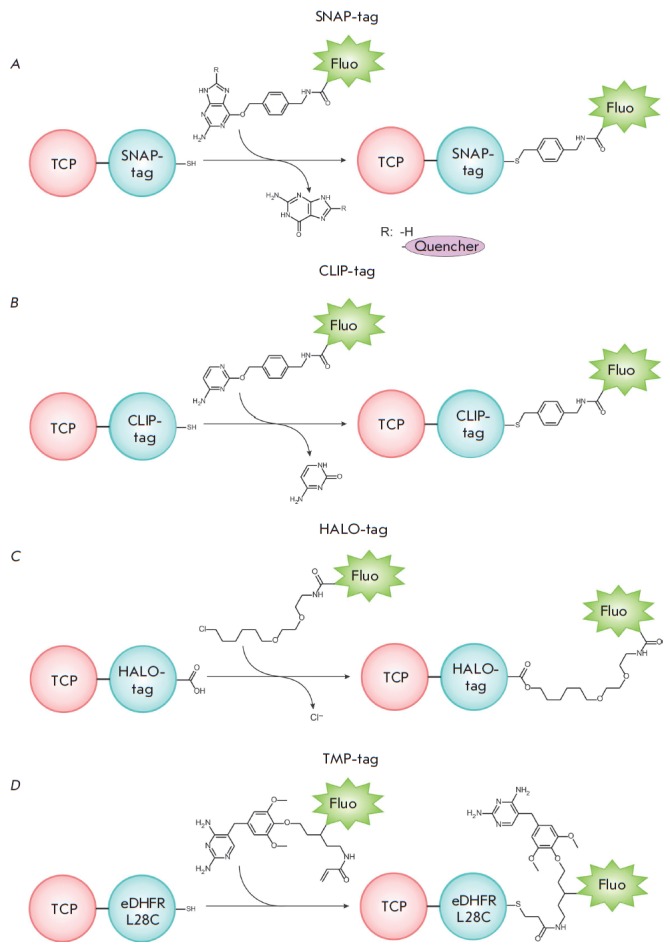
Enzymatic reactions for TCP labeling using SNAP-tag (A), CLIP-tag (B), Halo-tag (C), and TMP-tag (D)

In the SNAP-tag method, O^6^-alkylguanine transferase (AGT, *Fig. 11A*) acts as a fusion protein. AGT has a molecular weight of 20 kDa; it transfers alkyl groups from O^6^ of the alkylated guanine residue to the cysteine residue in the active site of the enzyme (see review [113]). Incubation of cells expressing AGT-TCP with the O6-benzylguanine substrate, in which the *p*-benzyl group carries a fluorophore, results in fluorescent labeling of AGT–TCP at cysteine in the active site of AGT [9]. Mutant forms of AGT were also obtained, catalyzing the reaction of alkyl radical transfer to AGT– TCP 50 times faster compared to the wild-type enzyme [107]. The SNAP-tag technology is currently the most commonly used to label intra- and extracellular proteins.

The CLIP-tag method is similar to SNAP-tag; it employs the mutant form of AGT, whose substrates are fluorescent analogues of O_2_-benzylcytosine (*Fig. 11B*) [108]. Despite the similarity between these technologies, SNAP-tag and CLIP-tag are characterized by different substrate specificities and can be used for simultaneous imaging of several cellular objects.

In the HALO-tag method, a genetically engineered variant of haloalkane dehalogenase acts as a fusion protein that specifically reacts with halogenated alkanes covalently bound to fluorophore (*Fig. 11C*) [114, 115]. This reaction, in which covalent binding is formed between the enzyme and the fluorescently labeled alkane, is highly specific and allows one to quickly insert a tag into proteins both *in vitro *and *in vivo *(10^3^–10^6^ M^−1^ s^−1^) under physiological conditions; importantly, this reaction is irreversible.

In all the methods mentioned above, the unreacted tag needs to be thoroughly washed out of the cells to achieve high contrast. SNAP-tag fluorogenic substrates containing an enzymatically removable fluorescent quencher were synthesized to eliminate this drawback (*Fig. 11A*). The enzymatic reaction with SNAP-tag results in cleavage of the quenching group, thus increasing the fluorescence intensity by more than fiftyfold. The advantage of these no-wash fluorophores was demonstrated using the spatio-temporal dynamics of epidermal growth factor receptors during cell migration [116].

The TMP-tag carrying a mutant of dihydrofolate reductase (eDHPR L28C) from *Escherichia coli *(molecular weight of ~18 kDa) is an alternative system. As a result of the enzyme reaction, fluorescently labeled 2,4-diamino-5-(3,4,5-trimethoxybenzyl)pyrimidine (trimethoprim, or TMP) binds to eDHPR–TCP (*Fig. 11D*) that is expressed in animal cells. The system is characterized by a relatively low background fluorescence and rapid kinetics [111]. A nonfluorescent TMP derivative containing a fluorophore and the corresponding quenching agent was produced to further reduce the background fluorescence caused by either the unbound or nonspecifically bound fluorescent tags. The TMP ligand binds to eDHPR–TCP, and the quencher is removed during the enzyme reaction. This method was shown to be efficient in labeling histones in the nuclei of HEK 903T cells [117].

## CONCLUSIONS

The methods for imaging biomolecules in living systems using synthetic fluorophores have been significantly modified in recent years. Various experimental and conceptual limitations have been overcome, primarily, for the site-directed reactions that allow one to insert a fluorescent tag into TCP. Modern technologies based on novel photoswitchable fluorophores are developing rapidly, such as subdiffraction microscopy that allows one to visualize cellular objects at resolutions in the nanometer scale.

Bioorthogonal labeling has made it possible to insert synthetic fluorophores that are much smaller than fluorescent proteins into TCP. The internal sites of TCP can be labeled using this method, as opposed to labeling N- and C-terminal regions when using FPs. Furthermore, the spectral properties of synthetic fluorophores can be easier tuned compared to FPs. Synthetic fluorophores can also be used to label non-protein objects (nucleotides, lipids, glycans, metabolites, etc.).

Although the constructs used in the enzyme methods for fluorophore insertion (SNAP-tag, CLIP-tag, HALO-tag, and TMP-tag) are of a size comparable to that of FPs, any small molecules can be inserted into TCPs using these techniques. Enzyme methods for fluorophore insertion into TCPs have been used more and more routinely to solve complex problems in modern biology and medicine. The use of these technologies in transgenic animals has been reported [118].

Today, the method involving the formation of metal– chelate complexes and sulfides is also frequently used for *in vivo *fluorescent labeling. As opposed to the previous techniques, it employs a small peptide fragment fused to TCP. New fluorophores exhibiting higher binding affinity and fluorescence intensity have been designed since the first publication that reported on the use of FlAsH. TCPs labeled with photoswitchable fluorescent tags have also been created using the metal–chelate technologies.

Taking into account the large number of application problems existing in the imaging of biomolecules in living systems, is it unlikely that a single universal fluorophore that meets all the desirable requirements can be designed. Moreover, the investigation of complex systems with several target objects requires the simultaneous use of several different fluorophores. Hence, further advance in this field solely depends on the synthesis of novel fluorophores that comply with the requirements of fluorescent microscopy, such as high photostability, low phototoxicity during long-term imaging, and the possibility of labeling multiple objects in living systems.

## Abbreviations

TCP: target cellular protein
FP: fluorescent protein
AGT: O6-alkylguanine transferase
eDHFR: dihydrofolate reductase
TMP: trimethoprim
DAPI: 4′,6-diamidino-2-phenylindole
NBD: 4-nitrobenz-2-oxa-1,3-diazole
dansyl chloride: 5-dimethylaminonaphthalene-1-sulfonyl chloride
EDANS: 5-((2-aminoethyl)amino)naphthalene-1-sulfonic acid
FRET: Förster resonance energy transfer
SRh 101: sulforhodamine 101
BODIPY: 4,4-difluoro-4-bora-3a,4a-diaza-s-indacene
FITC: fluorescein isothiocyanate
λex: excitation maximum
λem: emission maximum
ε: extinction coefficient
Φ: quantum yield
